# Susceptibility of Multidrug-Resistant Bacteria, Isolated from Water and Plants in Nigeria, to Ceragenins

**DOI:** 10.3390/ijerph15122758

**Published:** 2018-12-06

**Authors:** Marjan M. Hashemi, Augusta O. Mmuoegbulam, Brett S. Holden, Jordan Coburn, John Wilson, Maddison F. Taylor, Joseph Reiley, Darius Baradaran, Tania Stenquist, Shenglou Deng, Paul B. Savage

**Affiliations:** 1 Department of Chemistry and Biochemistry, Brigham Young University, Provo, UT 84602, USA; marjan.mhashemi@gmail.com (M.M.H.); bholden222@yahoo.com (B.S.H.); jccoburn2@gmail.com (J.C.); johnmartinwilson@gmail.com (J.W.); maddifaytaylor@gmail.com (M.F.T.); joseph.reiley@hotmail.com (J.R.); darius.baradaran@gmail.com (D.B.); tnance99@aol.com (T.S.); shengloudeng@hotmail.com (S.D.); 2 Department of Microbiology, University of Calabar, Calabar PMB 1115, Nigeria; oluchidinma@yahoo.com

**Keywords:** ceragenin, multidrug-resistant bacteria, biofilm, antimicrobial peptides, colistin

## Abstract

The continuous emergence of multidrug resistant pathogens is a major global health concern. Although antimicrobial peptides (AMPs) have shown promise as a possible means of combatting multidrug resistant strains without readily engendering resistance, costs of production and targeting by proteases limit their utility. Ceragenins are non-peptide AMP mimics that overcome these shortcomings while retaining broad-spectrum antimicrobial activity. To further characterize the antibacterial activities of ceragenins, their activities against a collection of environmental isolates of bacteria were determined. These isolates were isolated in Nigeria from plants and water. Minimum inhibitory concentrations (MICs) and minimum bactericidal concentrations (MBCs) of selected ceragenins and currently available antimicrobials against these isolates were measured to determine resistance patterns. Using scanning electron microscopy (SEM), we examined the morphological changes in bacterial membranes following treatment with ceragenins. Finally, we investigated the effectiveness of ceragenins in inhibiting biofilm formation and destroying established biofilms. We found that, despite high resistance to many currently available antimicrobials, including colistin, environmental isolates in planktonic and biofilm forms remain susceptible to ceragenins. Additionally, SEM and confocal images of ceragenin-treated cells confirmed the effective antibacterial and antibiofilm activity of ceragenins.

## 1. Introduction

The discovery and widespread use of antibiotics was one of the most important advances in medicine. These drugs were heralded for their effectiveness, and, as a result, began to be prescribed across the world. However, widespread use of antibiotics has resulted in the generation of mutational resistance in bacteria as well as identification of adaptational resistance mechanisms. These have led to the rise of hyper-resistant bacteria, often called superbugs [1,2]. Today, the phenomenon of antibiotic resistance has become a global public health concern, with 700,000 deaths across the globe each year attributed to antimicrobial resistance. This count is expected to reach 10 million by 2050 as the decreasing effectiveness of available market drugs continues to compound this problem [3]. Of particular concern is the widespread use of antimicrobial agents in food animals, which may be a major source of antimicrobial resistance that can spread drug-resistant pathogens to humans directly or through the environmental pollution of farm effluents [4].

**Figure 1:**
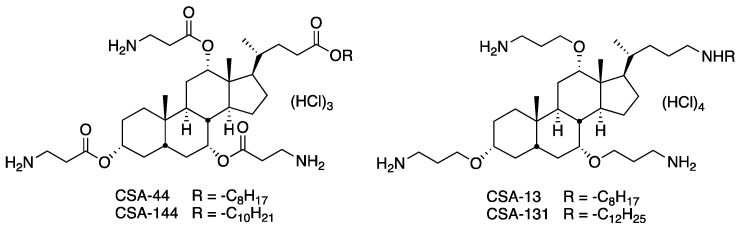
Structures of ceragenins CSA-44, CSA-144, CSA-13 and CSA-131.

Endogenous antimicrobial peptides (AMPs) are a key component of the body’s innate immune system, which is critical in fighting bacteria, fungi, and lipid-enveloped viruses. AMPs are typically cationic and amphiphilic in nature, which facilitates targeted association with negatively-charged pathogenic membranes, causing membrane disruption and cell death [5,6]. Interestingly, evidence has shown that bacteria are unable to achieve high levels of resistance to AMPs, making this an important area of antimicrobial research. However, AMPs can be expensive to manufacture synthetically and can be degraded in the presence of bacterial and host proteases [7,8]. In order to circumvent these challenges, ceragenins were developed from a common bile acid as non-peptide mimics of AMPs. Structure of ceragenins are shown in Figure 1. Ceragenins are cationic and amphiphilic, giving them analogous antimicrobial properties to AMPs. They are relatively inexpensive to produce and have shown potent activity against a broad spectrum of organisms. Of particular note is that ceragenins are active against methicillin-resistant *Staphylococcus aureus* [9], colistin-resistant *Klebsiella pneumoniae* [10], and fluconazole-resistant *Candida albicans* [11] and *Candida auris* [12]. To date, no bacteria have been shown to achieve high levels of resistance to ceragenins [13,14]. Ceragenins appear to be well tolerated in tissues and exhibit both the antimicrobial and secondary properties that are characteristic of many AMPs. Because of their promising therapeutic properties, ease of production, and possible synergistic effects, ceragenins represent an important target of study for further clinical development [15,16,17]. In this study, the antimicrobial resistance patterns of ten Nigerian bacterial strains isolated from the environment were determined by selected ceragenins and compared to commonly used antibiotics. The effects of ceragenins on the cell membranes of these isolates were observed by scanning electron microscopy (SEM). Additionally, we assessed the potential of selected ceragenins to eradicate biofilms formed by multidrug-resistant environmental isolates.

## 2. Materials and Methods

Ceragenins CSA-13, CSA-131, CSA-44, and CSA-144 were synthesized from a cholic acid scaffolding as previously described [18]. Colistin, chlorhexidine, kanamycin, polymyxin B, erythromycin, tetracycline, vancomycin, and ampicillin were purchased from Sigma–Aldrich (St Louis, MO, USA).

### 2.1. Isolation and Maintenance of Bacterial Isolates

Bacteriological analyses of water samples, including heterotrophic plate count (HPC), fecal coliform (FC) and total coliform count (TCC), were determined using both the direct pour plate method and membrane filtration techniques. No serial dilution was carried out. Nutrient agar medium was used for heterotrophic bacteria plate counts. MacConkey agar (MCA) was used for total coliform counts, and membrane fecal coliform (MF-C) agar medium was used for fecal coliform counts. The plates were inoculated in triplicate. Inoculations for HPC and TCC were conducted by adding 1 mL of sample to each plate. For membrane filtration, 100 mL of each water sample was filtered through a 0.45 µm membrane filter before aseptic transfer of the membrane onto MF-C agar or MCA for FC and TCC respectively. No dilution was used in either method. MCA was used for isolation of lactose fermenters (coliforms).

For isolates taken from plants, natural rubber latex (1 mL) was added to sterile distilled water (9 mL) and then serially diluted using a sterile micropipette. One gram of deteriorated rubber latex was added to sterile distilled water (100 mL) in a conical flask. The combination was mixed well, and an aliquot from this mixture was serially diluted in water (10^−1^ to 10^−10^ dilution). Selected dilutions were then used for the inoculation of agar plates. Each sample was plated in triplicate using the pour plate method. Inoculated plates were incubated for 24 h at 35–37 °C. Colonies were enumerated using a colony counter for total heterotrophic bacteria and total coliform counts. Discrete colonies were sub-cultured onto fresh nutrient agar plates aseptically to obtain pure cultures of the isolates and were stored in a refrigerator at 4 °C for further identification.

### 2.2. Identification of Isolates

Bacterial isolates were characterized based on microscopic appearance, colony morphology, gram staining reactions, and appropriate biochemical tests based on Bergey’s Manual of Determinative Bacteriology and as described by Cheesbrough [19]. The isolates were identified by comparing their characteristics with those of known taxa, as described by Cruickshank et al. [20] and Holt [21]. Table 1 shows the source of ten isolates used in this study.

### 2.3. Susceptibility Testing

Minimum inhibitory concentrations (MICs) were determined using a broth microdilution method in a 96-well microdilution plate according to the Clinical Laboratory Standards Institute protocol [22]. Briefly, 96-well plates were prepared with individual wells containing doubling concentrations of selected ceragenins, including CSA-13, CSA-44, CSA-131, and CSA-144 in the appropriate culture medium for a total volume of 100 µL. A selection of commercial antimicrobials including chlorhexidine, kanamaycin, colistin, polymyxin B, erythromycin, tetracycline, vancomycin and ampicillin was also used for comparison. An inoculation of 100 µL at 10^6^ CFU/mL was added to each well. Each of the 10 isolates was tested in duplicate and each plate contained positive and negative controls. Plates were incubated for 24 h at 37 °C. After incubation, results were obtained by examining wells for turbidity. Minimum bactericidal concentrations (MBCs) were determined by taking 10 μL from each well and plating on agar media. The MBC was defined as the lowest concentration of an antibacterial agent giving no visible colonies after 24 h incubation at 37 °C [10].

### 2.4. Scanning Electron Microscopy (SEM)

To observe the effect of ceragenins on cell membranes, selected isolates were cultured to mid-log phase and washed three times with PBS. Bacteria were re-suspended in PBS (OD_600_ = 0.2). CSA-131 (25 μg/mL) was then added and the mixtures were incubated at 37 °C for 1 h. A control was prepared by incubating the bacterial suspension without adding CSA-131. After collection via centrifugation, cells were washed with PBS three times. Gluteraldehyde (2.5% (w/v)) was added to fix the cells at 4 °C overnight. Resulting material was washed five times with PBS at 5000 rpm for 10 min using a microhematocrit centrifuge (Hettich Mikro 20, Hettich, Tuttlingen, Germany) to remove the glutaraldehyde. Osmium tetroxide (0.5 mL) was used as a second fixative reagent, and samples were stored at room temperature under a protective laboratory hood system for 2–3 h. Cells were washed with PBS five times at 14,000 rpm for 8 min. A graded ethanol series including 10%, 30%, 50%, 70%, 90% (1 time), 100% (3 times) and HMDS (2 times) for 15 min each was used to dehydrate the cells. Samples were collected by centrifuge each time and the supernatant was discarded after each centrifugation. Finally, dried bacterial specimens were sputter-coated with 5–10 nm of a Gold-Palladium alloy and visualized under a scanning electron microscope (FEI Helios NanoLab 600 SEM/FIB, Hillsboro, Oregon, USA) [12].

### 2.5. Biofilm Study Using XTT Assay

Biofilms of each isolate were grown for 48 h in separate wells in 96-well plates. Planktonic cells were then removed by washing three times with PBS. The biofilm-containing wells were treated with CSA-131 (100 µg/mL) and incubated for another 24 h. After another PBS wash, 100 µL of a mixed solution of 0.5 mg/mL 2,3-bis (2-methoxy-4-nitro-5-sulfophenyl)-5-[(phenylamino) carbonyl]-2H-tetrazolium hydroxide (XTT) and 10 mM menadione in acetone was added to each well. Plates were covered in aluminum foil and incubated for 2 to 3 h at 37 °C. Remaining solution was removed from each well and remaining dye in the wells was then quantified with a microtiter plate reader at 490 nm. Optical density results of test wells were compared with controls to determine the percent of biofilm remaining in each [23].

### 2.6. Confocal Laser Scanning Microscopy

Biofilms of *Acetobacter* spp. were formed on glass slides by complete submersion of slides in inoculated media (50 mL) and incubation for 48 h. Selected slides were treated with CSA-131 (100 µg/mL) and slides were further incubated at 37 °C for 24 h. Following incubation, glass slides were carefully removed from the solutions and rinsed three times with PBS. Using protocols of a BacLight Live/Dead Viability Kit (L13152, Molecular Probes, Inc), biofilms were stained and further imaged by a confocal laser scanning microscope (Olympus FluoView FV1000) at ×60 magnification [12].

## 3. Results and Discussion

### 3.1. Susceptibility of Isolated Bacteria

In an initial set of experiments, the MICs of several common antimicrobials against ten bacterial isolates were determined (Table 2). Gram-positive bacteria, including *Actinomycins* species, were relatively susceptible to chlorhexidine, vancomycin and ampicillin; however, all tested Gram-negative isolates showed very high MICs with selected antimicrobials, including chlorhexidine, kanamycin, colistin, polymyxin B, erythromycin and tetracyclin. For example, the MIC of *Enterococcus* species with chlorhexidine, kanamycin, colistin, polymyxin B, erythromycin and tetracyclin was 64, 100, >100, 100, 8 and 32 µg/mL, respectively. Of particular note is the MIC of colistin, which was more than 100 µg/mL with most of isolates tested. Colistin is generally considered the therapeutic of last resort for multidrug-resistant Gram-negative bacterial infections, so the presence of highly colistin-resistant isolates in this study is alarming.

To determine the activity of ceragenins against multidrug-resistant isolates, the MICs of selected ceragenins CSA-13, CSA-44, CSA-131 and CSA-144 were measured. These results are shown in Table 3. Ceragenins retained activity against all multidrug-resistant strains and showed low MICs compared to the commonly used antimicrobials. The MIC of CSA-13 and CSA-131 of 1–2 µg/mL with all of the highly multidrug-resistant isolates was of particular note. This result is consistent with our previous studies showing that ceragenins are highly active against methicillin-resistant *Staphylococcus aureus* [9] and colistin-resistant *Klebsiella pneumoniae* [10].

To confirm that the activity of ceragenins is bactericidal, MBCs of the same ceragenins were measured with the multidrug-resistant isolates. All tested ceragenins were found to be bactericidal at a range of 1–100 µg/mL, exhibiting bactericidal activity against strains such as *Pseudomonas* and *Actinomyces* spp. at the same concentrations as the corresponding MICs, suggesting that antibacterial activity of ceragenins are likely bactericidal rather than bacteriostatic.

### 3.2. Scanning Electron Microscopy (SEM)

**Figure 2:**
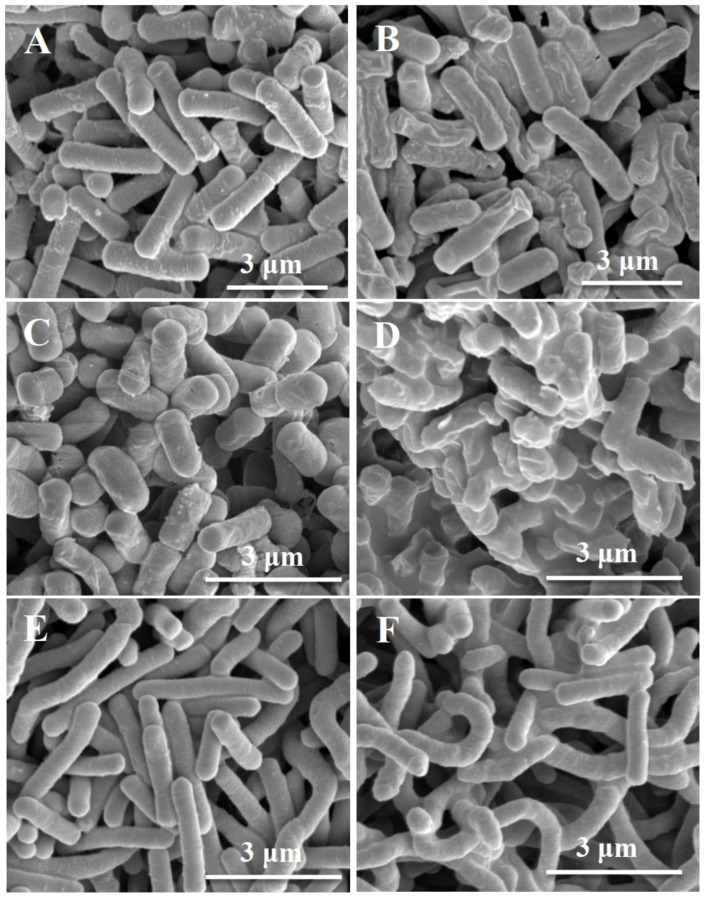
Scanning electron photomicrograph of untreated (**A**) and treated (**B**) *Klebsiella pneumoniae*, untreated (**C**) and treated (**D**) *Moraxella* spp., untreated (**E**) and treated (**F**) *Legionella pneumophila* with 25 μg/mL CSA-131.

To visualize the effect of ceragenins on the cell membrane, selected isolates were treated with a lead ceragenin, CSA-131, and their morphology was studied via SEM. Scanning electron photomicrographs of *Klebsiella pneumoniae*, *Moraxella* and *Legionella pneumophila* are shown in Figure 2. In the control without ceragenin treatment (Figure 2A,C,E), cells maintained normal morphology. In contrast, there were significant alterations in the morphology of cells treated with CSA-131 (Figure 2B,D,F). Treated cells are characterized by disruptions in the cell membrane along with increased roughness and wrinkling on the cell surface, confirming the membrane activity of ceragenins. Importantly, reported morphological changes in this study are consistent with previous reports describing changes in the structure of bacteria and fungi after treatment with ceragenins [24,25].

### 3.3. Determination of Susceptibility Profiles of Bacterial Biofilms

It is well established that biofilms have greater resistance to antimicrobials than planktonic cells [26]. There are several explanations for this increase in resistance. The extracellular matrix surrounding the cells in the biofilm prevents targeting and subsequent penetration by antimicrobials. The reduced growth rate of cells in biofilms, compared to planktonic cells, increases resistance to antimicrobials that target growth-specific factors. Other mechanisms include the inactivation or degradation of antimicrobials and efflux pumps that remove antimicrobials from the cells [27]. Despite these challenges, ceragenins have been shown to permeate the biofilm extracellular matrix, due to their relatively small size, and eradicate biofilms at relatively low concentrations. This activity is likely due to the mechanism of action of ceragenins, which is not dependent on the metabolic state of their targets [28].

**Figure 3:**
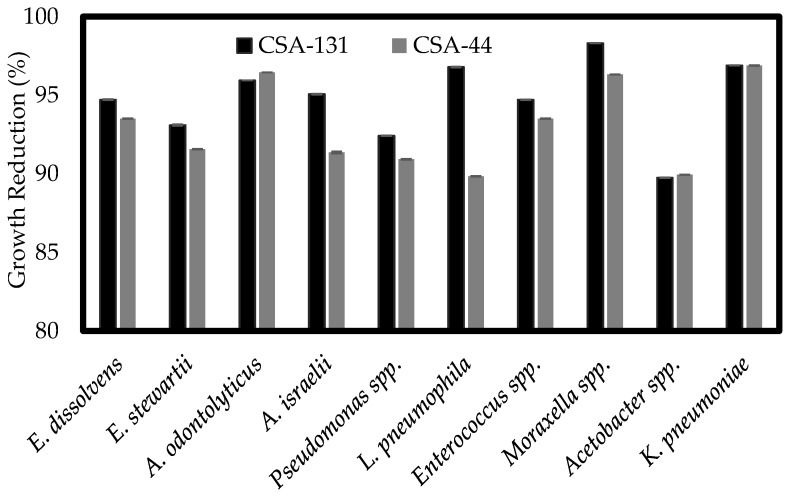
Reduction of established biofilms of ten isolates after 48 h incubation with CSA-131 or/and CSA-44 (100 μg/mL). Using the 2,3-bis (2-methoxy-4-nitro-5-sulfophenyl)-5-[(phenylamino) carbonyl]-2H-tetrazolium hydroxide (XTT) colorimetric based assay, metabolic activity of ceragenin-treated biofilms was measured and the percent of growth reduction was calculated in comparison to an untreated biofilm (control).

To quantify the impact of ceragenins on biofilm formation by multidrug-resistant isolates, an XTT assay was performed. The XTT assay measures metabolic activity of cells in the biofilm following a change in color. Corresponding biofilm growth reduction for each strain was calculated compared to a negative control that was not treated with any drugs. As shown in Figure 3, all representative ceragenins demonstrated strong antibiofilm activity against both Gram-positive and Gram-negative multidrug-resistant isolates and caused a substantial reduction of growth. Treatment of *Moraxella* spp., *K. pneumoniae* and *L. pneumophila* decreased biofilm mass by more than 96% compared to the negative control. A previous study showed that in a comparison of CSA-13 with ciproflaxicin, CSA-13 was shown to have greater activity against established biofilms formed by methicillin-resistant *S. aureus* [29].

### 3.4. Confocal Laser Scanning of Biofilms

**Figure 4:**
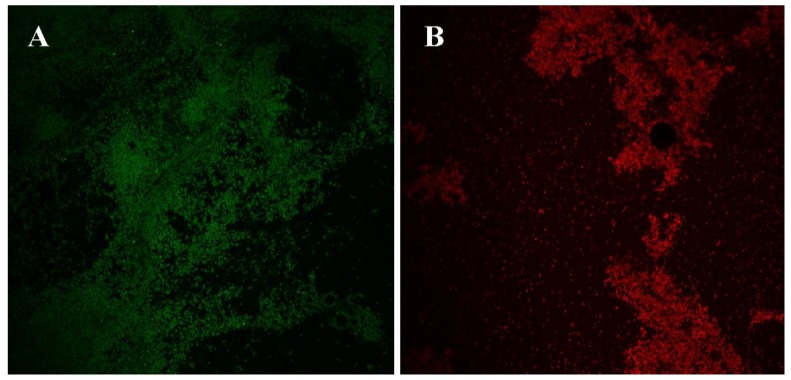
Confocal laser scanning micrographs (×60 magnification) of stained bacterial biofilms. Green: live cells; red: dead cells. (**A**) Untreated *Acetobacter* spp. (**B**) treated with CSA-131 (100 μg/mL).

To visualize antibiofilm properties of ceragenins, biofilms of *Acetobacter* spp. were treated with a lead ceragenin, CSA-131, and prepared for confocal microscopy. Confocal images are shown in Figure 4. In the images, a lack of biofilm is seen in some areas, which could be due to sample preparation in which slides were rinsed prior to staining to remove loosely adhered and planktonic organisms. Overall, as expected, untreated biofilms showed expected aggregates of live cells (green dye, Figure 4A), while ceragenin-treated biofilms exhibited comparable aggregates of dead cells (red dye, Figure 4B). Lack of biofilm was observed more often in the treated than in the untreated cells, which highlights ceragenins’ ability to destabilize established biofilms, facilitating their detachment from slide surfaces. Nagent, et al., [9] conducted a biofilm study using confocal microscopy and their images revealed that ceragenins efficiently penetrated established biofilms and led to cell death without significant alterations to the extracellular matrix. Additionally, a recent study demonstrated prolonged inhibition of biofilm formation on endotracheal tube surfaces when the tubes were coated with a CSA-131-containing hydrogel [30].

## 4. Conclusions

Obstacles to the development of novel antimicrobial agents include concerns that generation of resistance to one antimicrobial agent may result in cross-resistance to other antimicrobials. Since higher organisms have co-evolved with bacteria, the mechanisms by which they control bacterial growth may provide guidance for development of antimicrobial agents to which bacteria do not readily generate resistance. AMPs represent one of the key means by which higher organisms control bacterial growth. Ceragenins mimic key AMP structural features, specifically, multiple cationic (positive) charges juxtaposed with hydrophobic structure. The studies presented herein demonstrate that even highly multidrug-resistant environmental isolates largely remain susceptible to ceragenins. Additionally, previous studies showed that CSA-13 toxicity is comparable to LL-37 in tested human keratinocytes and it is not toxic to HatCat cells at bactericidal concentrations [31].

The ceragenins tested in this study gave MICs in the single µg/mL range in spite of the high MICs of commonly used antimicrobials, including the last resort antibiotic colistin. SEM images gave results comparable to earlier studies, demonstrating that ceragenins interact with bacterial membranes. Morphological changes to Gram-negative bacterial membranes are a hallmark of the activity of many AMPs, and we have shown, via transmission electron microscopy (TEM) and atomic force microscopy (AFM), that similar changes occur in bacterial membranes upon treatment with a ceragenin [24,32]. Further characterization of the lead ceragenins, CSA-131 and CSA-44, demonstrated that reduction of growth in a preformed biofilm was also successful; however, the extent of reduction was much less compared to the inhibition activity against planktonic cells. Confocal images verified the antibiofilm activity of ceragenins that occurs through penetration of the compound into the extracellular matrix of the biofilm.

Multidrug resistance in Nigeria is on the rise [33]. The highly resistant nature of the Nigerian environmental isolates analyzed in this study suggests that the careful designing and adoption of a multi-sectoral antimicrobial resistance surveillance plan for research and diagnostic purposes should be implemented. Relevant ministries and governmental agencies should consider the following: registration and observation of production premises, particularly where food-producing animals are concerned; improved biosecurity compliance in food-animal environments; banning antibiotic use for animal growth promotion or prophylactic treatment in animal husbandry; implementation of a drug withdrawal period for food animals.

## Figures and Tables

**Table 1:** Isolation source of bacteria used in this study.

Strains		Isolation Source
1	*Enterococcus* spp.	Rubber plant
2	*Actinomyces odontolyticus*	Rubber plant
3	*Actinomyces israelii*	Rubber plant
4	*Acetobacter* spp.	Rubber plant
5	*Moraxella* spp.	Rubber plant
6	*Enterobacter dissolvens*	Water
7	*Pseudomonas* spp.	Rubber plant
8	*Klebsiella pneumoniae*	Water
9	*Legionella pneumophila*	Water
10	*Erwinia Stewartii*	Water

**Table 2:** Minimum inhibitory concentrations (MICs) (μg/mL) of ten isolates with common antibiotics.

Strains	Chl	Kan	Col	Pol B	Ery	Tet	Van	Amp
*Enterobacter dissolvens*	8	64	>100	100	2	2	nm	nm
*Erwinia stewartii*	8	2	100	100	1	2	nm	nm
*Enterococcus* spp.	64	100	>100	100	8	32	nm	nm
*Pseudomonas* spp.	32	16	>100	>100	16	8	nm	nm
*Klebsiella pneumoniae*	32	32	>100	100	16	1	nm	nm
*Acetobacter* spp.	32	32	16	8	32	16	nm	nm
*Moraxella* spp.	64	64	>100	100	32	32	nm	nm
*Legionella pneumophila*	64	64	32	16	8	4	nm	nm
*Actinomyces odontolyticus*	4	nm	nm	nm	nm	nm	1	1
*Actinomyces israelii*	4	nm	nm	nm	nm	nm	2	2

Chl: chlorhexidine; Kan: kanamycin; Col: colistin; Pol B: polymyxin B; Ery: erythromycin; Tet: tetracycline; Van: vancomycin; Amp: ampicillin. nm: not measured.

**Table 3:** Comparison of the MIC (minimum bactericidal concentrations (MBC)) (μg/mL) of ten isolates to selected ceragenins.

Strains	CSA-13	CSA-44	CSA-131	CSA-144
*Enterobacter dissolvens*	1(8)	2(10)	2(8)	2(10)
*Erwinia stewartii*	2(8)	4(10)	2(8)	4(10)
*Actinomyces odontolyticus*	1(1–2)	1(1)	1(1)	2(2)
*Actinomyces israelii*	2(4)	1(2)	2(4)	2(4)
*Pseudomonas* spp.	1(1)	4(4)	1(1)	4(4)
*Legionella pneumophila*	8(8)	4(8)	4(4)	16(32)
*Enterococcus* spp.	16(32)	8(8)	4(32)	32(100)
*Moraxella* spp.	10(32)	4(16)	4(16)	24(100)
*Acetobacter* spp.	2(32)	4(64)	2(32)	4(64)
*Klebsiella pneumoniae*	2(4)	4(8)	2(4)	4(4)
